# Supplementary material for: Cytoplasmic male sterility and abortive seed traits generated through mitochondrial genome editing coupled with allotopic expression of atp1 in tobacco
Source: Front Plant Sci. 2023 Sep 15;14:1253640. doi: 10.3389/fpls.2023.1253640 (PMC10541219; doi:10.3389/fpls.2023.1253640)
Supplement: Supplementary file 1 [file DataSheet_1.zip › Figures.S1-S13.docx]

**(A)**

**atg**gaactttctccccgagctgcggaactaacaagtctattagaaagtcgaattagcaacttttacaccaattttcaagtggatgagatcggtcgagtggtctcagttggagatgggattgcacgtgtttatggattgaacgagattcaagctggggaaatggttgaatttgccagcggtgtgaaaggaatagccttgaatcttgagaatgagaatgtagggattgttgtctttggtagtgatactgctattaaagaaggagatcttgtcaagcgcactggatctattgtggatgttcctgcgggaaaggctatgctagggcgtgtggtcgatggcttgggagtacctattgatggaaggggggctctaagcgatcacgagcgaagacgtgtcgaagtgaaagcccctggtattattgaacgtaaatctgtgcacgagcctatgcaaacagggttaaaagcggtagatagcctggttcctataggtcgtggtcaacgagaacttataatcggggaccgacaaactggaaaaactgctattgctatcgataccatattaaaccaaaagcaactgaactcaagggccacctctgagagtgagacattgtattgtgtctatgtagcgattggacagaaacgctcaactgtggcacaattagttcaaattctttcagaagcgaatgctttggaatattctattcttgtagcagccaccgcttcggatcctgctcctctacaatttttggccccatattctgggtgtgccatgggggaatatttccgcgataatggaatgcacgcattaataatctatgatgatcttagtaaacaggcggtagcatatcgacaaatgtcattattgttacgccgaccaccaggtcgtgaggctttcccaggggatgttttctatttacattcccgtctcttagaaagagcggctaaacgatcggaccagacaggcgcaggtagcttgaccgccttacccgtcattgaaacacaggctggagacgtatcggcctatattcccaccaatgtgatccccattactgatggacaaatctgtttggaaacagagctcttttatcgcggaattagacctgcgattaacgtcggcttatctgtcagtcgcgtcgggtctgccgctcagttgaaaactatgaaacaagtctgcggtagttcaaaactggaattggcacaatatcgcgaagtggccgcccttgctcaatttggctcagaccttgatgctgcgactcaggcattactcaatagaggtgcaaggctgacagaagtaccgaaacaaccacaatatgcaccactgccaattgaaaaacaaattctagtcatttatgcagctgtcaatggattctgtgatcgaatgccactagacagaatttctcaatatgagagagccattccaaatagtgtcaaaccagaattactacaatcctttttagaaaaaggtggcttaactaacgaaagaaagatggaaccagatacattcttaaaagaaagtgctttagcttttatt**taA**

**(B)**

**gtcgac**gagaggcggtttgcgtattggctagagcagcttgccaacatggtggagcacgacactctcgtctactccaagaatatcaaagatacagtctcagaagaccaaagggctattgagacttttcaacaaagggtaatatcgggaaacctcctcggattccattgcccagctatctgtcacttcatcaaaaggacagtagaaaaggaaggtggcacctacaaatgccatcattgcgataaaggaaaggctatcgttcaagatgcctctgccgacagtggtcccaaagatggacccccacccacgaggagcatcgtggaaaaagaagacgttccaaccacgtcttcaaagcaagtggattgatgtgaacatggtggagcacgacactctcgtctactccaagaatatcaaagatacagtctcagaagaccaaagggctattgagacttttcaacaaagggtaatatcgggaaacctcctcggattccattgcccagctatctgtcacttcatcaaaaggacagtagaaaaggaaggtggcacctacaaatgccatcattgcgataaaggaaaggctatcgttcaagatgcctctgccgacagtggtcccaaagatggacccccacccacgaggagcatcgtggaaaaagaagacgttccaaccacgtcttcaaagcaagtggattgatgtgatatctccactgacgtaagggatgacgcacaatcccactatccttcgcaagacccttcctctatataaggaagttcatttcatttggagaggacacgctgaaatcaccagtctctctctacaaatctatctctctcgagctttcgcagatctgtcgaacca**ggatcc**act**ATG**gcttctcggaggcttctcacctctctcctccgtcaatcggctcaacgtggcggcggtccaatttcccgatccttgggaaactccatccctaaatccgctgcacgcgcctcttcacgcgcgtcccctaagggattcctcttaaaccgcgccgtacagtacgctacctctgcagcagcacccgcatctcagccatccatggaactctcaccaagggcagcagaacttacttcactcctcgaatctagaatctcaaacttctacacaaactttcaggttgatgaaattggaagagttgtttcagttggagatggtattgctagggtttatggacttaatgagattcaagctggtgaaatggttgagtttgcttctggagttaagggtatcgctttgaatcttgaaaatgagaacgttggaatcgttgtttttggttcagatactgctattaaggagggagatttggttaaaagaacaggttctattgttgatgttccagctggaaaagctatgcttggtagagttgttgatggattgggtgttcctattgatggaaggggtgctttgtctgatcatgaaagaaggagagttgaggttaaggctccaggaattattgaaagaaaatcagttcatgagcctatgcaaactggtcttaaggctgttgattctttggttccaattggaaggggtcaaagagaacttattattggagataggcaaactggaaagactgctatcgctatcgatacaatccttaaccaaaagcaattgaattcaagagctacttctgaatcagagacattgtattgtgtttacgttgctattggacaaaaaaggtctactgttgctcaacttgttcaaattttgtctgaagctaatgctcttgagtattcaattttggttgctgctacagcttctgatccagctcctcttcaatttttggctccatattcaggatgcgctatgggtgaatactttagagataatggtatgcatgctcttattatatatgatgatttgtcaaagcaagctgttgcttacagacaaatgtctcttttgcttagaagaccaccaggaagggaagcttttcctggagatgttttctatcttcattctaggttgcttgagagagctgctaaaaggtctgatcaaactggagctggttcacttacagctttgccagttattgagactcaagctggagatgtttcagcttacatccctactaacgttatctctatcacagatggacaaatttgtcttgaaacagagttgttttacaggggtattagaccagctattaatgttggactttctgtttcaagagttggttcagctgctcaattgaagactatgaaacaagtttgcggttctttgaagcttgaattggctcaatatagggaggttgctgcttttgctcaatttggatcagatttggatgctgctactcaagctttgcttaataggggtgctagacttacagaagttttgaagcaacctcaatatgctccacttcctatcgagaagcaaattcttgttatatatgctgctgttaatggtttctgtgatagaatgccacttgataggatctctcaatacgaaagagctatcttgaactcagttaagcctgaattgcttcaatcttttcttgagaaaggaggtttgacaaacgagaggaagatggagctcgatacttttctcaaggagtccgcactcgcattcatc**TGAagatct***atagattataaacttctgtgactttcttttcttctctttgccaaaataatttagtttgtgacatcccggatttttttggaggaccaagaggtccagaattctggttttgttttacatccaatgcgagattatagagacatgcagccaagcctttgttgccagagacccccttttctgttatgtcacataataaagggggtaaatggtgatcttgtatatctgattttcaagtctttttcgagaattttggattccctgattatcaaatgcctttctgaaacgctttctttctatatgtggttaacttcacgtccattttatccattctgccttgaaagcttcaatgtaataggagcagttaatatgctaagcggatacaaatcatatttcttggcgcgacttaattttattgcagtatgaatacttgtagaaaatatgagtatttgactaagcttataagcggcaaaccagcttataagtcacttttactttacttcatctacgcgtttggtaaaattaaaagtgttaagtctagtgcttgtaagcttttaagtcttaagttgtcataagttggtcacatctaaatttgaactgcccctgcccccatcttccccaaaagaatctgcattcacgctaagaggttatgcaaactctttagggctaaagaattgtcgtcaaattataactaaagccaaagttctaaaatatgtcttacagagataatgtctagagaataataagatttatatgcagagacaattcctttagattcctttaggatgataataacacacgccagacatacattatagtggaaaaaataatgaacaggaaattacacccaataaccttgaacatatatggtcttgattttttgattctttgtccc***ggtacc**

**Figure S1. DNA sequences of the native mitochondrial *atp1* gene and the repurposed 35S:nATP1 construct designed to function as a nuclear gene. (A)** DNA sequence of the tobacco *atp1* gene. The start and stop codons are in bold, capitalized text. The six ‘c’ nucleotides shaded grey represent locations where cytidines are converted to uridines in the mature transcript by RNA editing. The 22 bp recognition sites of the ARCUS enzymes ATP-5/6 and ATP-7/8 are shaded green and turquoise, respectively. The shared 4 bp 3’ overhangs produced after cleavage are underlined. **(B)** Sequence of construct 35S:nATP1. An enhanced CaMV 35S promoter sequence is shaded yellow. Sequences encoding the tobacco ATP2 transit peptide (plus the first 12 codons of the mature protein) are highlighted in magenta. The 1530 bp sequence in lower case unshaded type represents the custom-synthesized nATP1 reading frame codon optimized for expression in the nucleus of tobacco. The italicized sequences highlighted in green correspond to the region downstream of the tobacco *ATP2* stop codon. Within the green highlighted region, sequences that are underlined can be found in *ATP2* cDNAs, and thus represent the minimal extent of the *ATP2* 3’-UTR. The start and stop codons of the nATP1 open reading frame are indicated in bold, capitalized type. Restriction sights that were engineered to facilitate cloning are shown in lower case, bold type. *ATP2 s*equences encoding the transit peptide and the 3’-UTR were obtained from GenBank accession NCAA01008649.


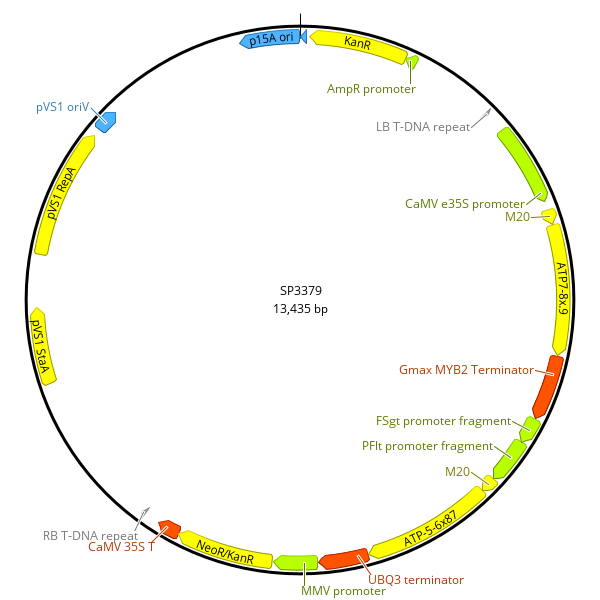


**Figure S2. MitoARCUS vector SP3379.** ATP 5-6x87 and ATP 7-8x.9 encode ARCUS enzymes designed to target two distinct 22 bp sequences in *atp1*. M20, transit peptide from putative Arabidopsis HNH endonuclease (GenBank accession KAG7646785). ATP 5-6x87 is under the transcriptional control of a chimeric *Figwort Mosaic Virus – Peanut Chlorotic Streak Caulimovirus* promoter; termination of transcription is mediated by the termination sequences of the soybean *Ubiquitin3* (UBQ3) gene. ATP 7-8x.9 is under the transcriptional control of an enhanced CaMV 35S promoter; termination of transcription is mediated by the termination sequences of the soybean *MYB2* gene. Expression of the *nptII* selectable marker gene (NeoR/KanR) is driven by a *Mirabilis Mosaic Virus* (MMV) promoter and terminated by the CaMV 35S terminator region. RB, right border; LB, left border.

**Figure S3. Semi-quantitative PCR analysis of K326 haploid plants transformed with 35S:nATP1.** The tobacco *Actin-7* gene (GenBank accession XM_016658880) was used as a control. PCR reactions were conducted for 18 cycles and 21 cycles for nATP1 and *Actin-7*, respectively.

**Figure S4. Transient expression of ARCUS-GFP fusion proteins in tobacco protoplasts.** ARCUS-GFP fusions were introduced with: (a) no localization peptide; (b) a nuclear localization signal (NLS); and (c) the transit peptide M20 from a putative Arabidopsis HNH endonuclease (GenBank accession KAG7646785). Pictures were taken at 20X and 40X magnification (top and bottom, respectively). M20 transit peptide sequence: MGSSFSASFTNSTTAAAVPPPSPPSSPSRSNVKSNGEERPRF

**Figure S5. Primer combinations designed to amplify *atp1.*** Primer sequences and sizes of predicted amplification products are shown in Table S1. F1 = atp1_F1; F2 = atp1_F2; F3 = atp1_F3; R1 = atp1_R1; R2 = atp1_R2; R3 = atp1_R3. M, molecular weight marker. EV, empty vector.


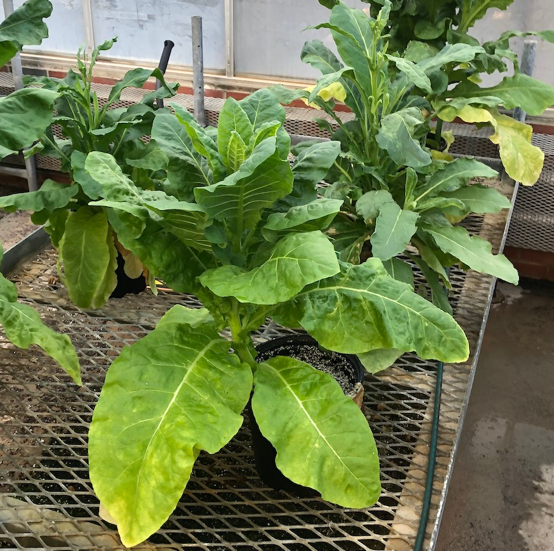
**
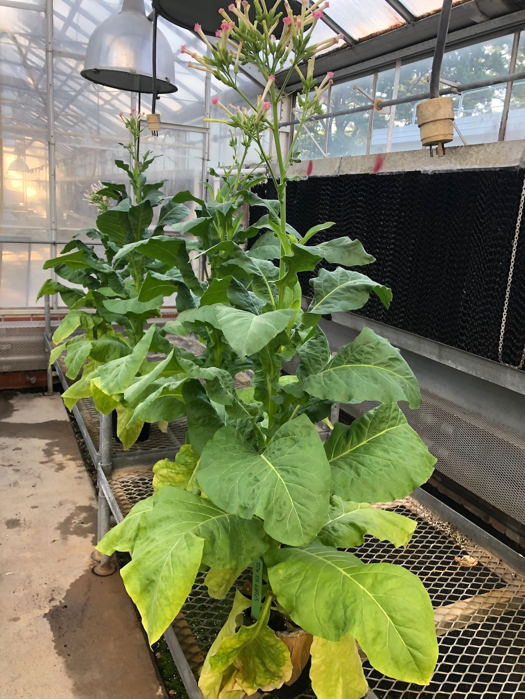
**

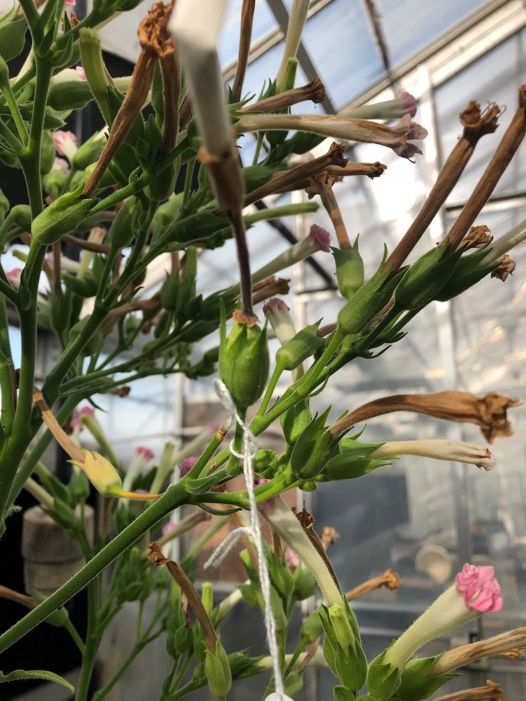


**Figure S6. Examples of mature T_0_ 35S:nATP1/Δ*atp1* plants.** Representative 35S:nATP1/Δ*atp1* plants that display abnormal **(A)** or typical **(B)** growth patterns are shown 10 weeks after transplanting to soil. **(C)** Ovary/capsule development is observed in flowers that have been cross-pollinated. Fertilized flower has a paper pollination tube and is tagged with a string at the base.

**Figure S7. Examples of normal and 35S:nATP1/Δ*atp1* flowers and anthers.** Empty vector control (K326 EV#13) and 35S:nATP1/Δ*atp1*#1 whole flowers **(A)** and anthers **(B)** are shown at Stage 12, when dehiscence normally occurs. Prior to being photographed, tobacco anthers were tapped on the black background to reveal the presence or absence of pollen.


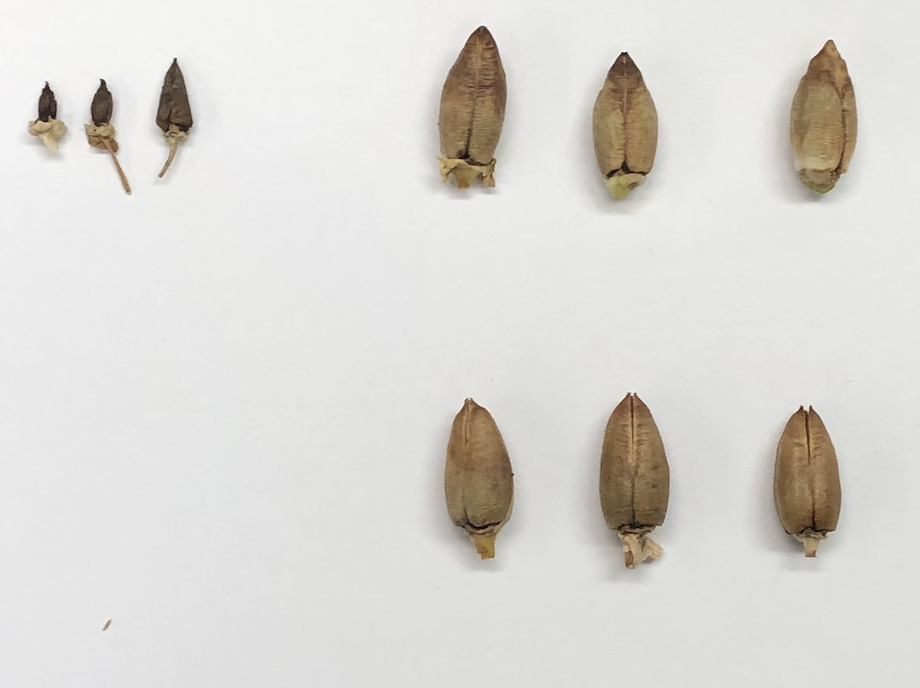


**(A)**

Unfertilized

35S:nATP1/Δ*atp1*#8

Cross-fertilized

35S:nATP1/Δ*atp1*#8 X WT K326

Self-fertilized WT K326


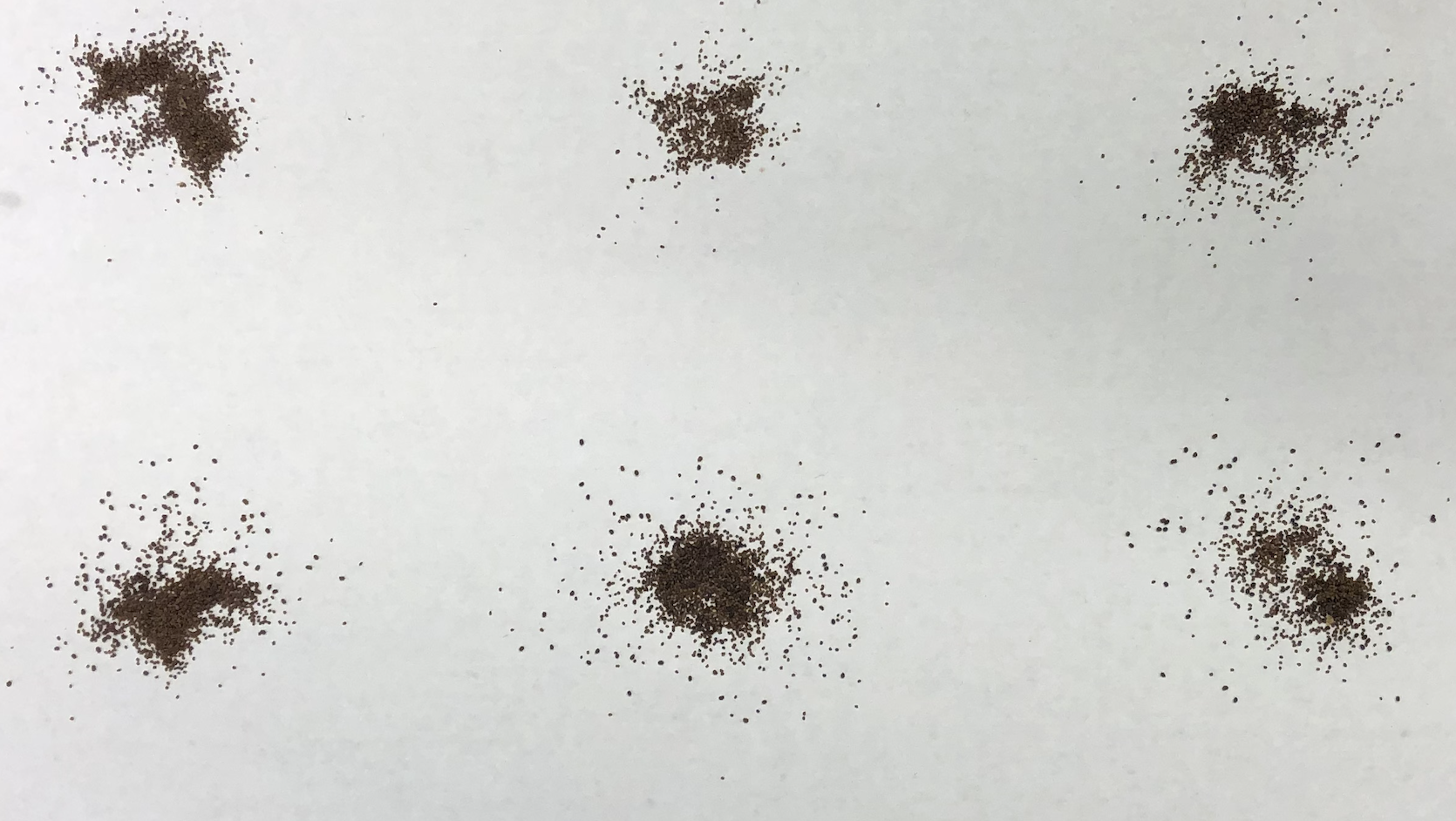


**(B)**

35S:nATP1/Δ*atp1*#8 X WT K326

WT K326

**Figure S8. Mature capsules (A) and seeds (B) of plant 35S:nATP1/Δ*atp1*#8 fertilized with WT tobacco pollen.**

**Figure S9. Plants used for PacBio sequence analysis.** Tissue culture [tc] was used to propagate T_0_ events 35S:nATP1/Δ*atp1*#22, #8 and #16. Plants derived from the original T_0_ event 35S:nATP1/Δ*atp1*#16 through culturing displayed a stunted growth phenotype similar to the parental plant.

**Figure S10. Circos plots of contigs corresponding to the mitochondrial genomes of 35S:nATP1/Δ*atp1*#8 (A), 35S:nATP1/Δ*atp1*#16 (B), and 35S:nATP1/Δ*atp1*#22 (C) aligned to the tobacco mitochondrial reference genome BA000042.** Blocks of sequence that align between the contigs and reference genome are connected by arcs of the same color. Contigs that end with ‘tr’ were manually trimmed to remove NUMT sequences located at the 5’ or 3’ ends. The location of *atp1* on the reference genome is indicated on each plot.

**Figure S11. PCR and DNA sequence validation of predicted recombination events in plants possessing Δ*atp1* mutations. (A)** Recombination junctions 5’ and 3’ of *atp1* in 35S:nATP1/Δ*atp1*#8. **(B)** Recombination junctions 3’ of *atp1* in 35S:nATP1/Δ*atp1*#16 and 35S:nATP1/Δ*atp1*#22. **(C)** Junctions of pre-existing subgenomic species created by recombination across a 148 bp fragment between *cox3* and *atp1* and the interior of Rep2. Numbering is in accordance with reference genome BA000042. Portions of the DNA chromatograms derived from sequence analysis of the unique PCR products shown in the adjacent gels that validate the recombination junctions predicted in PacBio-derived contigs are shown. For the PCR experiments shown in **(C)**, the *atp6-*specific primers were diluted 1:10 (1 μM final concentration) to prevent the smaller PCR product from outcompeting the amplification of the much larger alternative PCR product.

**Figure S12. SSS in 35S:nATP1/Δ*atp1*#8 X 35S:nATP1 F_1_ progeny.** Semi-quantitative PCR was conducted using primers specific for the two subgenomic variants shown in Figure S11C. The tobacco *atp6* gene was used as a control. All PCR reactions were run for 25 cycles.

**Figure S13. 35S:nATP1/Δ*atp1*#8 X 35S:nATP1 F_1_ plants at flowering.**
