# Supplementary material for: Cytoplasmic male sterility and abortive seed traits generated through mitochondrial genome editing coupled with allotopic expression of atp1 in tobacco
Source: Front Plant Sci. 2023 Sep 15;14:1253640. doi: 10.3389/fpls.2023.1253640 (PMC10541219; doi:10.3389/fpls.2023.1253640)
Supplement: Supplementary file 1 [file DataSheet_1.zip › Tables S1-S3.docx]

**Table S1. PCR primers and reaction conditions used in this study**

| Name | Sequence (5’-3’) | Purpose | Product  Size | Annealing  Temp/time | Extension  Temp/time |
| --- | --- | --- | --- | --- | --- |
| ATP2TP_F  ATP1_R | actATGgcttctcggaggcttctc  TCAGATGAATGCGAGTGCGGA | Semi-quantitative PCR of 35S:nATP1 T_0_ individuals | 1731bp | 65^o^C/30 sec | 72^o^C/2 min |
| NtAct_F4  NtAct_R2 | AGCACCCTGTTCTTCTCAC  GTCAAGCTCCTGCTCGTAG | *Actin7* amplification | 385bp | 59^o^C/30 sec | 72^o^C/40 sec |
| atp1_F1  atp1_R1 | tgagatcggtcgagtggtct  tacgtctccagcctgtgttt | *atp1* amplification | 928bp | 64^o^C/15 sec | 72^o^C/30 sec |
| atp1_F1  atp1_R2 | tgagatcggtcgagtggtct  actgacagataagccgacgt | *atp1* amplification | 1042bp | 64^o^C/15 sec | 72^o^C/30 sec |
| atp1_F1  atp1_R3 | tgagatcggtcgagtggtct  TAAGGCGGTCAAGCTACCTG | *atp1* amplification | 898bp | 63^o^C/15 sec | 72^o^C/30 sec |
| atp1_F2  atp1_R1 | ggaactttctccccgagctg  tacgtctccagcctgtgttt | *atp1* amplification | 1011bp | 64^o^C/15 sec | 72^o^C/30 sec |
| atp1_F2  atp1_R2 | ggaactttctccccgagctg  actgacagataagccgacgt | *atp1* amplification | 1125bp | 64^o^C/15 sec | 72^o^C/30 sec |
| atp1_F2  atp1_R3 | ggaactttctccccgagctg  TAAGGCGGTCAAGCTACCTG | *atp1* amplification | 980bp | 65^o^C/15 sec | 72^o^C/30 sec |
| atp1_F3  atp1_R1 | CAAGTGGATGAGATCGGTCG  tacgtctccagcctgtgttt | *atp1* amplification | 936bp | 63^o^C/15 sec | 72^o^C/30 sec |
| atp1_F3  atp1_R2 | CAAGTGGATGAGATCGGTCG  actgacagataagccgacgt | *atp1* amplification | 1050 bp | 63^o^C/15 sec | 72^o^C/30 sec |
| atp1_F3  atp1_R3 | CAAGTGGATGAGATCGGTCG  TAAGGCGGTCAAGCTACCTG | *atp1* amplification | 906bp | 63^o^C/15 sec | 72^o^C/30 sec |
| atp6_F1  atp6_R3 | atccgggccttaatccttgc  tgcgaggggaaaacttttgt | *atp6* amplification | 511 bp | varied | varied |
| hptII_F  hptII_R | GTGTACGCCCGACAGTCCCGGC  CCCGATTCCGGAAGTGCTTGAC | *HPTII* amplification | 705bp | 63^o^C/20 sec | 72^o^C/15 sec |
| nptII_F  nptII_R | GAACAAGATGGATTGCACGC  AGAAGGCGATAGAAGGCGAT | *NPTII* amplification | 773 bp | 63^o^C/15 sec | 72^o^C/15 sec |
| E10_F  E10_R | CAGCAGATAGTGACGCTAACC  AACCCTGGATCAAGTGTGCG | *CYP82E10* amplification | 549 bp | 63^o^C/15 sec | 72^o^C/15 sec |
| ARCUS_F1  ARCUS_R1 | CCAACCACGTCTTCAAAGCA  ATAGACCTCCCACGGATCCT | Amplification of SP3379 construct | 714 bp | 64^o^C/15 sec | 72^o^C/30 sec |
| #8 5’br_F3  #8 5’br_R4 | ATCGAACTTGAACTCCGGGT  CCAACAACTTCACTCCGGAG | Amplification of recombination junction at 5’ break point in event 35S:nATP1/Δ*atp1*#8 | 843 bp | 63^o^C/15 sec | 72^o^C/30 sec |
| #8 3’br_F2  #8 3’br_R3 | CCCTCAAAGCCCCATTAACG  AAGACCTAAGCTTGAAGTCAAG | Amplification of recombination junction at 3’ break point in event 35S:nATP1/Δ*atp1*#8 | 754 bp | 61^o^C/15 sec | 72^o^C/30 sec |
| #16/22 5’br:v1_F3  #16/22 5’br:v1_R1 | ATCGAACTTGAACTCCGGGT  ATCTTCACTTTACGCCACGC | Amplification of recombination junction at 5’ break point in event 35S:nATP1/Δ*atp1*#8 and #22 (variation 1) | 3250 bp | 64^o^C/20 sec | 72^o^C/2 min |
| #16/22 5’br:v2_F5  #16/22 5’br:v2_R4 | AGCGCAGTACTTCCGTAACT  TATCGCCCTTTGTTCCTCCA | Amplification of recombination junction at 5’ break point in event 35S:nATP1/Δ*atp1*#8 and #22 (variation 2) | 3214 bp | 64^o^C/20 sec | 72^o^C/2 min |
| #16 3’br_F1  #16 3’br_R2 | TATGGATAGGGCGACGTGAC  GCCAAGACTGTACGAGGAGA | Amplification of recombination junction at 3’ break point in event 35S:nATP1/Δ*atp1*#16 | 734bp | 64^o^C/15 sec | 72^o^C/30 sec |
| #22 3’br_F1  #22 3’br_R2 | GTTCGGTCAGGCTTGCTTAG  AGTAAGGCATTGGGGATCGT | Amplification of recombination junction at 3’ break point in event 35S:nATP1/Δ*atp1*#22 | 790 bp | 64^o^C/15 sec | 72^o^C/30 sec |

- All reactions were initiated with a 30 sec denaturation at 98^o^C for 30 sec, and terminated with a 7 min extension at 72^o^C after 33 cycles (or number of indicated cycles for semi-quantitative PCR assays).
- Reactions were conducted using Phusion Taq and 5x HF reaction buffer (New England Biolabs – cat. #M0530L).
- All denaturation steps were conducted at 98^o^C for 10 sec.
- Unless otherwise indicated, primers were added at a concentration of 10 μM.
- The *atp6* primers were amplified using the conditions preferred for amplification of the *atp1* primers they were paired with.

**Table S2. Summary of transformation experiments**

| Background transformed | Number of individuals recovered | Number of individuals with greatly diminished *atp1* amplification | Number of individuals that were PCR positive for the *nptII* selectable marker |
| --- | --- | --- | --- |
| 35S:nATP1 | 15 | 15 | 15 |
| EV Control* | 9 | 0 | 2 |

*EV Control represents K326 plants possessing only the pCAMBIA1300 vector.

**Table S3. PacBio sequence assembly summary**

| Line name | Total reads | N50 of Reads | Reads that map to Mt  genome | % Reads  mapping to Mt genome | Number of contigs in assembly* | Number of contigs that represent NUMTs* | Number of legitimate Mt genome contigs** |
| --- | --- | --- | --- | --- | --- | --- | --- |
| 35S:nATP1Δ*atp1*#8 | 878,706 | 14,113 | 40,862 | 4.7% | 94 | 89 | 5 |
| 35S:nATP1Δ*atp1*#16 | 543,041 | 14,286 | 18,282 | 3.4% | 40 | 36 | 4 |
| 35S:nATP1Δ*atp1*#22 | 911,783 | 13,168 | 44,784 | 4.9% | 95 | 90 | 5 |

*In addition to the mitochondrial genome, this includes contigs that share homology to the chloroplast genome, as the mitochondrial genome also contains fragments of the chloroplast genome

**A contig was classified as legitimate if the sequence displayed >99.99% nucleotide identity to the tobacco mitochondrial reference genome (BA000042) over its entire length, and an NUMT if it shared <98% identity to the reference genome over its length.

Mt, mitochondrial
